# Correlation Between Body Mass Index and Fibroadenoma

**DOI:** 10.7759/cureus.5219

**Published:** 2019-07-23

**Authors:** Mehreen K Bhettani, Mubarik Rehman, Humera N Altaf, Syed M Ahmed, Ahmad A Tahir, Muhammad S Khan, Tanzeel Imran

**Affiliations:** 1 General Surgery, Royal Oldham Hospital, Manchester, GBR; 2 General Surgery, Shifa College of Medicine, Islamabad, PAK; 3 Surgery, Shifa International Hospital, Islamabad, PAK; 4 General Surgery, University of Maryland Medical Center, Maryland, USA; 5 General Surgery, Leighton Hospital, Crewe, GBR; 6 General Surgery, Shifa Tameer E' Millat University, Shifa International Hospital, Islamabad, PAK; 7 Pathology, Jamila Sultana Foundation, Rawalpindi, PAK

**Keywords:** breast neoplasms, fibro adenoma, excision, obesity, surgery, breast cancer, body mass index, public awareness, adolescent

## Abstract

Introduction

Among all benign conditions, the fibroadenoma is the most common lesion worldwide as well as in Pakistan. Clinicians often face the dilemma of whether to remove the mass or to monitor it by means of periodic follow-up examinations. Although the removal of these lesions is a definitive solution, surgery may involve unnecessary excision of benign lesions and unbecoming cosmesis. Body mass index (BMI) is a known risk factor for the development of breast cancer.However, the relationship between BMI and benign breast diseases is still unclear. Some studies showed that increased BMI is a risk factor for benign breast diseases; however, a large number of studies showed that a decrease in BMI is the risk factor for benign breast diseases.

Material and methods

This was a descriptive cross-sectional study conducted at the Department of General Surgery, Pakistan Institute of Medical Sciences (PIMS), Shaheed Zulfiqar Ali Bhutto Medical University, Islamabad, Pakistan. All patients fulfilling inclusion criteria were assessed in the breast clinic of PIMS. A final diagnosis of fibroadenoma was made after a triple assessment. Weight in kilograms and height in meters were measured. All the information was recorded in a specifically designed proforma accordingly by the postgraduate trainee. BMI was calculated by the formula: BMI=Weight in kgs/height in meters. Other variables that were noted include patients' age, gender, contact number, and hospital visit. The data were analyzed using SPSS version 21 (IBM Corp., Armonk, NY, US).

Results

The BMI of study patients was 21.8 ± 1.3, ranging from 19 to 24.9. Out of 300 patients presenting with benign breast disease, 60 (20%) had a fibroadenoma while 240 (80%) had other benign breast diseases. Out of 136 patients with high BMI, 42 (30.8%) had a fibroadenoma while out of 74 patients with low BMI, eight (10.8%) had a fibroadenoma; however, out of 90 patients with normal BMI, 10 (11.1%) had a fibroadenoma. Our study population showed an increased risk of fibroadenoma formation in the adolescent age group with an OR value of 8.54 (CI 4.38-16.63, P<0.001). There were also additional statistical correlations between higher BMI and the site of the lesion being the upper outer quadrant of the breast (t= 4.326 P<0.01). There was no significant correlation of BMI with size and increased number of lesions (P=0.280 and P=0.175).

Conclusion

High BMI seems to be a substantial risk factor for the development of a fibroadenoma, particularly in young adolescent females.

## Introduction

Benign breast disease is the most common cause of breast problems presenting in breast clinics [1]. Globally, benign pathological states account for 90% of the clinical presentations related to the breast [2]. Up to 30% of women will suffer from benign breast disease, requiring treatment at some time of their lives [1]. Benign breast diseases consist of a spectrum of diseases, from physiological swelling and tenderness to palpable lump, nodules, or infections [3]. To address the undue anxiety in both patients and clinicians, the term aberration of normal development and involution was coined by Cardiff Breast Clinic [1]. Among all benign conditions, the fibroadenoma is the most common lesion worldwide and in Pakistan [4]. The clinicians often face the dilemma of whether to remove the mass or to monitor it by means of periodic follow-up examinations. Although the removal of these lesions is a definitive solution, surgery may involve unnecessary excision of benign lesions and unbecoming cosmesis [5]. Moreover, a policy of conducting surgery on all patients with fibroadenomas would place an enormous burden on healthcare systems [5]. A balanced and rational approach to the management of fibroadenoma of the breast needs to address crucial questions about its association with breast cancer, especially whether or not it is a marker of increased risk of breast malignancy. Another consideration to be weighed is that a substantial percentage of these lesions undergo spontaneous regression [6]. Therefore, it is imperative that the risk factors for the development of these lesions must be identified and addressed.

Body mass index (BMI) is a known risk factor for the development of breast cancer [7]. However, the relationship between BMI and benign breast diseases is still unclear. Some studies showed that increased BMI is a risk factor for benign breast diseases; however, a large number of studies showed that a decrease in BMI is a risk factor for benign breast diseases [8].

In a study, Abbas H reported that women of pubescent and reproductive age with low BMI <25 have a greater chance of developing fibroadenoma (100%) than women having BMI >25 (67%) [9].

In their study, Shesh Kumar et al. showed that most benign breast disease (BBD) patients were young and premenopausal or perimenopausal. Many studies have demonstrated an inverse relationship between BMI and BBD, i.e. increasing BMI had a lower incidence of BBD [10].

Despite multiple publications on the relation of fibroadenoma and BMI in international literature, there are few studies in Pakistan that showed a correlation between BMI and fibroadenoma. Therefore, it is the need of the hour to ascertain any correlation in our population.

## Materials and methods

This was a descriptive cross-sectional study conducted at the Department of General Surgery, Pakistan Institute of Medical Sciences, Shaheed Zulfiqar Ali Bhutto Medical University, Islamabad, Pakistan. The study was conducted after taking approval from the hospital research ethics committee and was completed six months after the approval of the synopsis, from 24-03-2016 to 23-09-2016.

All patients diagnosed with benign breast diseases between the ages of 13 to 35 years were included in the study. Patients with single or multiple lumps were included in the study.

All patients with fibroadenoma more than 5 cm in size, having a family history of breast cancer, or having any other malignancy and other co-morbid conditions like chronic renal failure, chronic liver disease, thyrotoxicosis, and tuberculosis were excluded from the study.

Informed written consent for the study was taken before the start of the study. All patients were assessed clinically and a thorough examination was performed in the outpatient department. A final diagnosis of fibroadenoma was made after the triple assessment. Weight in kilograms and height in meters were measured. All the information was recorded on a specifically designed proforma accordingly by the postgraduate trainee. BMI was calculated by the formula: BMI=Weight in kgs/height in meters [2]. Other variables that were noted included the patient’s age, gender, contact number, and hospital visit.

The data were analyzed by SPSS version 21 (IBM Corp. Armonk, NY, US). Descriptive statistics were calculated for all variables like age and BMI. Mean and standard deviation was calculated for quantitative variables like age, BMI, and size of fibroadenoma along with frequency and percentage. Effect modifiers like age, site, size, and number of fibroadenomas were controlled by stratification. The post-stratification chi-square test was applied. A p-value of less than 0.05 was considered significant.

## Results

In this study, 300 female patients having a benign breast disease were enrolled to determine the frequency of fibroadenoma and its correlation with BMI. The mean age of the study patients was 23.05 ± 4.17 years, ranging from 13 to 35 years. The majority of the study patients, almost (74%), were below 30 years of age. Around 26% were above 30 years. The average size of fibroadenoma was 2.5 ± 1.90 cm in width, ranging from 1 cm to 5 cm.

Data regarding anthropometric parameters, i.e. weight, height, and BMI, were calculated as mean and standard deviation. The average weight of study patients was 63.5 ± 5.8 kilograms, ranging from 52 to 76 kg. The average height was 169.5 ± 13.0 cms. The BMI of study patients was 21.8 ± 1.3, ranging from 19 to 24.9. Out of 300 patients presenting with benign breast disease, 60 (20%) had a fibroadenoma while 240 (80%) had other benign breast diseases.

Out of 136 patients with high BMI, 42 (30.8%) had a fibroadenoma while out of 74 patients with low BMI, eight (10.8%) had a fibroadenoma; however, out of 90 patients with normal BMI, 10 (11.1%) had a fibroadenoma.

Out of 166 patients, 39 (23%) with a mass or lesion in the upper outer quadrant had fibroadenomas; out of 79 patients with a mass or lesion in the lower outer quadrant, six (8%) patients had fibroadenomas; nine out of 39 patients (23%) having masses or lesions in the upper inner quadrant had fibroadenomas; and six out of 16 patients (37.5%) having masses or lesions in the lower inner quadrant had fibroadenomas.

Thirty-nine (39) out of 95 patients (41%) having age 13-20 years had fibroadenomas, 12 out of 127 patients (5%) having age 21-30 years had fibroadenomas, and nine out of 78 patients (12%) having age 31-35 years had fibroadenomas. Table 1 shows the distribution of BMI across various categories of lesion sizes, 19 out of 55 patients (41%) having lesion size less than 1 cm had fibroadenomas, 26 out of 137 patients (19%) having lesion size 1 cm to 3 cm had fibroadenomas, 15 out of 108 patients (12%) having lesion size 3 cm to 5 cm had fibroadenomas.

**Table 1:** Distribution of BMI across various categories of sizes of lesions BMI: body mass index

Size of Lesion					Total	P-value
		Less Than One Cm	One To Three Cm	Three To Five Cm		
Body Mass Index	high	35	48	53	136	0.28
	low	17	34	23	74	
	normal	3	55	32	90	
Total		55	137	108	300	

Out of 157 patients with a single breast lesion, 41 (26.1%) had fibroadenoma. Out of 93 patients with two breast lesions, 09 (9.6%) had a fibroadenoma, whereas, in 38 patients with three breast lesions, nine (23.6%) had a fibroadenoma. Similarly, one (8.3%) out of 12 patients with four breast lesions had a fibroadenoma. There was no significant correlation of BMI with size and increased number of lesions (P = 0.280 and P = 0.175) (Table 2).

**Table 2:** Distribution of BMI across various categories of numbers of lesions BMI: body mass index

		No Of Lesions				Total	P-value
		One	Two	Three	Four		
Body Mass Index	High	79	30	26	1	136	0.175
	Low	36	25	10	3	74	
	Normal	42	38	2	8	90	
Total		157	93	38	12	300	

Risk estimation and statistical analysis suggested an OR value of 3.57 for high BMI as a risk factor of developing fibroadenoma (CI 1.68 - 7.57 P < 0.01) while low BMI did not show a significant association with fibroadenomas ( OR 0.96 CI 0.36 - 2.59 P = 0.95 ) (Table 3).

**Table 3:** Fibroadenomas in different BMI groups BMI: body mass index

BMI	Number of Patients with Fibroadenoma		Total Number of Patients Total	%	Odds Ratio	CI	P-value
High BMI	YES	NO	136	45.3%	3.57	1.68 – 7.57	0.01
	42	94					
Normal BMI	10	80	90	30.1%	1.12	0.34 – 3.1	0.15
Low BMI	8	66	74	24.6%	0.95	0.36 – 2.59	0.95

**Figure 1:**
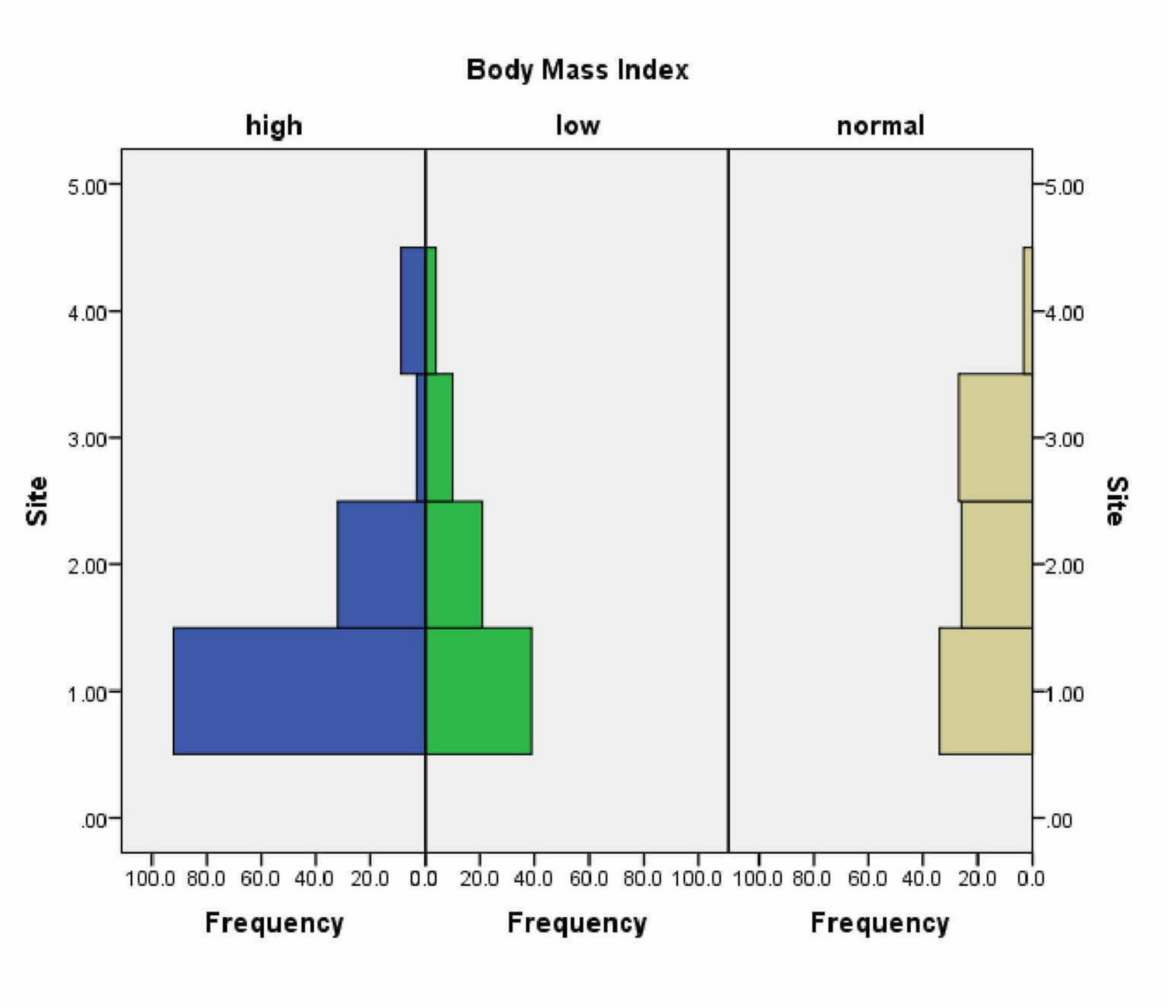
Correlation of BMI with the site of lesion (p<0.05), (1 = UOQ, 2 = LOQ, 3 = UIQ, 4 = LIQ) BMI: body mass index; UOQ: upper outer quadrant; LOQ: lower outer quadrant; UIQ: upper inner quadrant; LIQ: lower inner quadrant

Our study population showed an increased risk of fibroadenoma formation in the adolescent age group with an OR value of 8.54 (CI 4.38 - 16.63 P < 0.001). There were also additional statistical correlations between higher BMI and the site of the lesion being the upper outer quadrant of the breast (t=4.326 P < 0.01) (Figure 1).

## Discussion

Increased BMI is one of the rapidly rising problems surfacing across the globe, leading to socioeconomic and medical implications [11]. The correlation of increased BMI and the development of breast cancer is well-established; however, its correlation to benign breast disease is still under study [12]. Moreover, excess body weight significantly increases the risk of numerous diseases and clinical disorders, including all-cause mortality, coronary and cerebrovascular diseases, various cancers, type-2 diabetes mellitus, hypertension, liver disease, and asthma, as well as psychopathology, among others [13]. The relationship of BMI with benign breast disease is less clear. Certain benign pathologies are associated with increased BMI while some of the benign breast diseases are associated with decreased body mass index [14]. The significance of management of breast disease is two-fold: it is increasingly being presented in clinics due to public awareness and advancements in the identification and control of the associated risk factors [15].

According to the literature, all benign breast pathologies were noted to decrease with increasing BMI, except for fibroadenoma, which peaked in the BMI group from 25 kg/m^2^ to 29.9 kg/m^2^ [15]. The presence of benign pathologies was associated with age as expected. Interestingly, although BMI is associated with increased risk of breast cancer, increasing BMI was not associated with the benign pathologies that are associated with increased risk of breast cancer [16]. In our study, out of 136 patients with high BMI, 42 (30.8%) had fibroadenoma while out 74 patients with low BMI, eight (10.8%) had fibroadenoma; however, out of 90 patients with normal BMI, 10 (11.1%) had fibroadenoma.

Another study conducted by Okoth C et al. concluded that BMI is directly related to benign breast disease. In this study, 195 women with benign breast lumps were included. Benign proliferative breast disease was noted in 35 patients (18%). Benign proliferative breast disease with atypia was recorded in 11 (5.6%) patients. The mean age and BMI were 28.4 years and 23.26 kg/m^2^, respectively [17]. The most common lesion found was fibroadenoma in 111 patients (57%) [16]. This was comparable to the results of our study.

In contradiction to this, authors have reported an inverse relationship between benign breast disease and BMI [18]. Studies have shown high BMI is associated with histopathologic patterns of active fibroadenomas reflecting estrogenic stimulation; therefore, concluding that increased BMI is a significant risk factor of fibroadenoma formation [19].

A study by Schnitt SJ et al., which found the prevalence of benign proliferative breast disease in Japan was as high as 18% among women younger than 40 years [20]. A similar finding was documented in the North American study, the prevalence of benign breast disease in high BMI patients was 23% and low BMI patients was 17% [21].

Limitations of this study include small sample size and lack of region data on anthropometrics and incidence of begin breast disease. A larger study group and a multicenter study would provide further validity to the results of the study. Therefore, further studies are needed, testing this correlation on a larger sample size in a wider population to further elucidate the correlation of BMI to the incidence of fibroadenoma.

## Conclusions

High BMI seems to be a substantial risk factor for the development of fibroadenoma, particularly in young adolescent females. The most common site of lesion in our population was the upper outer quadrant of the breast. There was no significant association of BMI with the size and quantity of lesions. Females with high BMI should be encouraged to resume a lifestyle such as to keep their BMI within the normal range in order to avoid the development of fibroadenoma among other associated morbidities.
